# Verschwörungstheorien und paranoider Wahn: Lassen sich Aspekte kognitionspsychologischer Modelle zu Entstehung und Aufrechterhaltung von paranoiden Wahnüberzeugungen auf Verschwörungstheorien übertragen?

**DOI:** 10.1007/s11757-022-00710-2

**Published:** 2022-04-14

**Authors:** Stephanie Mehl

**Affiliations:** 1 Klinik für Psychiatrie und Psychotherapie, Universität Marburg, Rudolf-Bultmann-Str. 8, 35039 Marburg, Deutschland; 2 Fachbereich Soziale Arbeit und Gesundheit, Frankfurt University of Applied Sciences, Nibelungenplatz 1, 60318 Frankfurt am Main, Deutschland

**Keywords:** Kontinuumshypothese, Wahn, Kognitive Bias, Voreiliges Schlussfolgern, Externale Kontrollüberzeugungen, Delusions, Continuum hypothesis, Cognitive bias, Jumping to conclusions, External locus of control

## Abstract

Der vorliegende narrative Übersichtsartikel stellt zunächst verschiedene Definitionen von Verschwörungstheorien und Verschwörungsmentalität vor und präsentiert Studien zu Zusammenhängen zwischen Persönlichkeitseigenschaften, Symptomen psychischer Störungen und Verschwörungstheorien. Anschließend werden die Kontinuumshypothese des Wahns sowie neuere Konzeptualisierungen von allgemeinem und paranoiden Wahn diskutiert, des Weiteren werden typische kognitionspsychologische Modelle präsentiert, die die Entstehung und Aufrechterhaltung von Wahnüberzeugungen durch eine Interaktion von biologischen Vulnerabilitätsfaktoren, psychologischen Faktoren und sozialen Faktoren erklären. In diesen Modellen mediieren sowohl kognitive Verarbeitungsstile („cognitive biases“) als auch emotionale Prozesse die Entstehung und Aufrechterhaltung von Wahn, beispielsweise die Tendenz, voreilige Schlussfolgerungen zu treffen („jumping to conclusions bias“), sowie externale Kontrollüberzeugungen und ein externalisierender personalisierender Kausalattributionsstil. Anschließend wird diskutiert, ob Menschen, die Verschwörungstheorien zugeneigt sind, ebenfalls ähnliche kognitive Verarbeitungsstile aufweisen, die auch bei Personen zu finden sind, die paranoiden Wahnüberzeugungen zustimmen und unter psychotischen Störungen leiden. Parallelen bestehen zwischen beiden Personengruppen beispielsweise im Hinblick auf eine Neigung zu externalen Kontrollüberzeugungen und einen external personalisierenden Kausalattributionsstil. Auch bestehen Ähnlichkeiten in der Neigung, voreilige Schlussfolgerungen zu treffen. Fragen nach Gemeinsamkeiten und Unterschieden zwischen beiden Phänomenen sollten in weiteren präregistrierten experimentellen Studien quer- sowie längsschnittlich untersucht werden. Möglicherweise könnten moderne niedrigschwellige Interventionsmethoden der kognitiven Verhaltenstherapie von Psychosen (CBTp) in die Beratung oder Prävention von Verschwörungstheorien implementiert werden.

## Abstract (en)

In the present narrative review a set of definitions on conspiracy theories and conspiracy mentality are introduced. In addition, studies on associations between personality variables, symptoms of mental disorders and endorsement of conspiracy theories are discussed. With respect to delusions, the continuum hypothesis is put forward that assumes a continuum between subclinical and clinical symptoms of psychosis. In addition, models on the formation and maintenance of delusions/paranoid delusions are presented that assume an interaction between biological vulnerability, psychological and social factors that leads to the formation and maintenance of paranoid delusions. Cognitive biases and emotional processes mediate the formation of persecutory delusions, e.g. a tendency to jump to conclusions (jumping to conclusions bias) and an external personal attribution style. Regarding cognitive biases and emotional processes, similarities between persons with psychoses and persecutory delusions and persons who endorse conspiracy theories are discussed. Both groups are more prone towards an external locus of control and an external personal attribution stye and were both found to present a jumping to conclusion bias in comparison to nonclinical controls. It is discussed whether these similarities can be further explored using preregistered longitudinal and experimental studies. In addition, the potential of cognitive behavioral therapy for psychosis (CBTp) interventions and their implementation in the prevention and counselling of persons who endorse conspiracy theories and are motivated to reflect on or to change these theories is discussed.

## Verschwörungstheorien und Verschwörungsdenken und paranoide Wahnvorstellungen: Zusammenhänge und Unterschiede

Im Rahmen der aktuellen SARS-CoV-2-Pandemie sind Verschwörungstheorien und deren Anhänger:innen stark in den Fokus der gesellschaftlichen und wissenschaftlichen Debatte gerückt. In diesem Zusammenhang existieren viele verschiedene Verschwörungstheorien, die z. B. die Entstehung und Verbreitung des Virus unbekannten und sinistren Personen zuschreiben (Imhoff und Lamberty 2020a). Diese Theorien sind in sozialen und traditionellen Medien weit verbreitet worden. Die Zustimmung zu auf die Coronapandemie bezogenen Verschwörungstheorien stand bei vielen Beteiligten mit der Weigerung, sich an Gegen- und -Schutzmaßnahmen zu beteiligen, um die Weiterverbreitung des Virus zu reduzieren, in quer- und längsschnittlichem Zusammenhang (Metaanalyse von Bierwiaczonek et al. (o.J.); Pummerer et al. (2022)). Weitere querschnittliche Zusammenhänge bestanden zwischen coronabezogenen Verschwörungstheorien und Ängsten vor der Coronaschutzimpfung bzw. einer Weigerung, sich impfen zu lassen (Freeman et al. 2020; Jennings et al. 2021).

## Was sind Verschwörungstheorien?

Es besteht in der aktuellen Debatte, beispielsweise auf Twitter, große Unklarheit darüber, welche Theorien in den Rang von Verschwörungstheorien erhoben werden sollten. So werfen sich Anhänger:innen und Gegner:innen verschiedener Theorien gegenseitig vor, Verschwörungstheorien zu verbreiten, was in diesem Zusammenhang auch als exkludierende Kommunikationsstrategie angesehen werden kann.

Wissenschaftliche Kriterien für Kennzeichen einer Theorie als Verschwörungstheorie bietet beispielsweise Brotherton et al. (Brotherton et al. 2013):

- Die Behauptung über die Verschwörung ist nicht verifizierbar.
- Die Theorie ist nicht die wahrscheinlichste Erklärung für ein Ereignis; es können wahrscheinlichere Erklärungen identifiziert werden.
- Die Theorie ist oft aufsehenheischend oder sensationalistisch.
- Es werden sinistre und mächtige Akteure als Verursachende der Verschwörung angenommen.
- Die Theorie basiert auf nur geringer und nicht auf hinreichender Evidenz.
- Die Theorie isoliert sich selbst gegen ihre eigene Widerlegung (beispielsweise indem angenommen wird, dass sich die Theorie nicht prüfen lässt, da die Verschwörenden ihre Aufdeckung verhindern werden).

Interessanterweise bietet diese Definition einige Ähnlichkeiten zu einer Definition von Wahnüberzeugungen, deren Inhalte von Jaspers (1973) ebenfalls als „nichtrealisierbar“ angesehen wurden.

Gemäß Imhoff und Lamberty (Imhoff und Lamberty 2020b) ist eine solche Definition einer Verschwörungstheorie als eine Theorie, die sich einer Verifizierung wiedersetzt, allerdings problematisch, da es fast unmöglich ist, eine solche Theorie hinsichtlich ihres Wahrheitsgehalts und ihrer Plausibilität zu prüfen. Dies gilt ebenfalls für wahnhafte Überzeugungen, deren Plausibilitätsprüfung häufig auch für Diagnostiker:innen schwierig zu realisieren ist, sodass in diesem Bereich ebenfalls alternative Definitionen entwickelt wurden, die ohne eine Plausibilitätsprüfung der Wahnüberzeugung auskommen.

Auf eine alternative Definition von Verschwörungstheorien hat sich die Taskforce Verschwörungstheorien der Deutschen Gesellschaft für Psychologie (Imhoff et al. 2021) geeinigt: „Von einer Verschwörungstheorie spricht man, wenn Menschen glauben, dass ein Ereignis durch geheime Absprachen einer Gruppe von Personen zustande gekommen ist, und zwar zu deren Vorteil und dem Schaden der Allgemeinheit.“

Von einer Einstufung einer Theorie als Verschwörungstheorie ist die generelle Tendenz abzugrenzen, sich die Welt generell über Verschwörungstheorien zu erklären, die „Verschwörungsmentalität“, die definiert ist als eine Einstellung, die die Wahrnehmung beinhaltet, dass die Welt durch im Geheimen ausgeheckte Pläne und Verabredungen einzelner kleiner Gruppen gekennzeichnet ist (Imhoff 2020; Imhoff und Bruder 2014).

Die vorliegende Diskussion weist darauf hin, dass die Wiederlegung von Verschwörungstheorien insgesamt als schwierig einzuschätzen ist (Lewandowsky et al. 2012; Uscinski et al. 2016) und bestimmte Persönlichkeitseigenschaften die Wahrscheinlichkeit erhöhen könnten, einer Verschwörungstheorie anzuhängen (Douglas et al. 2019).

Die Diskussion darüber, ob bestimmte Persönlichkeitseigenschaften dazu führen, dass Personen anfälliger für Verschwörungserzählungen werden und welche spezifischen psychologischen Mechanismen im Zusammenhang mit einem ausgeprägten Glauben an Verschwörungstheorien stehen, wird bereits seit den 90er-Jahren des letzten Jahrhunderts geführt. Viele Studien untersuchten Zusammenhänge zwischen bestimmten Persönlichkeitseigenschaften und der Neigung, Verschwörungstheorien zuzustimmen.

Einige Studien wiesen nach, dass einige eher psychosenahe Persönlichkeitseigenschaften wie beispielsweise die Neigung, ungewöhnliche Wahrnehmungen zu erleben und paranoiden Wahnüberzeugungen zuzustimmen (Brotherton und Eser 2015; Swami et al. 2016a), und schizotype Persönlichkeitsmerkmale (Barron et al. 2014) mit der Neigung assoziiert sind, Verschwörungstheorien zuzustimmen. Aber auch Persönlichkeitsmerkmale wie erhöhte Ängstlichkeit (Greziak-Feldmann 2013) und eine Neigung, sich schnell zu langweilen (Swami et al. 2016a), standen mit der Zustimmung zu Verschwörungstheorien im Zusammenhang.

Auch narzisstische Persönlichkeitszüge und ein gleichzeitig niedriger Selbstwert waren in Regressionsanalysen mit einer Zustimmung zu Verschwörungstheorien assoziiert, und der Zusammenhang wurde durch zusätzlich bestehende paranoide Wahnüberzeugungen mediiert (Cichocka et al. 2016). In einer Metaanalyse über 96 Primärstudien wurden Zusammenhänge zwischen den 5 wichtigsten Persönlichkeitseigenschaften Verträglichkeit, Offenheit für neue Erfahrungen, Gewissenhaftigkeit, Introversion und Neurotizismus zusammengefasst, jedoch konnten hier keine signifikanten Zusammenhänge nachgewiesen werden (Goreis und Voracek 2019). Insgesamt weisen die Befunde auf mögliche Zusammenhänge hin, allerdings ist anzumerken, dass viele Studien in diesem Bereich nicht präregistriert wurden und es sich auch jeweils um Zufallsbefunden handeln könnte.

## Wahnüberzeugungen: Kontinuumsmodelle

Jaspers (1973) beschrieb als Gemeinsamkeiten von Wahnideen die außergewöhnlich Überzeugung, mit der an ihnen festgehalten wird, die Unbeeinflussbarkeit durch Erfahrungen oder Argumente anderer Menschen sowie die Nichtrealisierbarkeit des Inhalts bei einem bizarren Wahn. Wie bereits diskutiert, beinhaltet diese Definition ebenfalls das Problem, dass eine Überprüfbarkeit sich schwierig gestalten kann.

Vor dem Hintergrund der Jaspers’schen Wahn-Definition ging man lange Zeit von qualitativen Unterschieden zwischen Wahngedanken und gewöhnlichen Gedanken aus. Zahlreiche Studien (Lincoln und Keller 2008; Lincoln et al. 2009; Nuevo et al. 2012; Peters et al. 1999) und Metaanalysen (van Os et al. 2009) zeigen jedoch, dass Wahnphänomene nicht nur bei Personen nachweisbar sind, die unter psychischen Störungen wie z. B. Schizophrenie, schizoaffektiven Störungen, wahnhaften Störungen oder anderen psychotischen Störungen leiden, sondern auch in der Normalbevölkerung vertreten sind. In Schätzungen liegt die Lebenszeitprävalenz von psychotischen Störungen in der Normalbevölkerung etwa bei 7 % (0,3–0,7 % für eine Schizophrenie, 0,3 % für eine schizoaffektive Störung und 0,2 % für eine wahnhafte Störung) (Linscott und van Os 2013). Gemäß der Kontinuumshypothese bilden subklinische und klinische Wahnphänomene ein quantitatives Kontinuum, und zwischen beiden besteht ein fließender Übergang.

**Figure 1:**
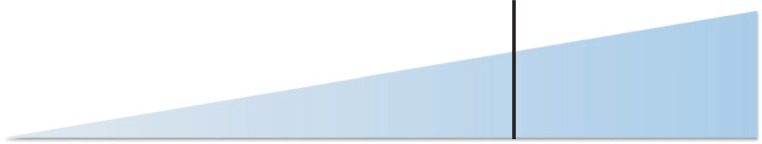

Aktuelle Definitionen für Wahn sind somit nicht einfach zu treffen, und die Abgrenzung eines wahnhaften Gedankens von „normalen“ Gedanken ist von verschiedenen kulturellen, individuellen Rahmenbedingungen und inhaltlichen Komponenten der Wahnüberzeugung abhängig (Abb. 1).

Neuere Konzeptualisierungen des Wahns gehen von einer multidimensionalen Struktur der wahnhaften Überzeugung aus (Freeman 2007), in der auch die 3 Dimensionen:

1. Überzeugungsstärke,
2. psychische Belastung durch die wahnhafte Überzeugung,
3. Beschäftigung mit oder Grübeln und Sorgen über die wahnhafte Überzeugung

eine wichtige Rolle spielen. Oltmanns (1988) postulierte in vergleichbarer Weise, dass eine Überzeugung umso eher als wahnhaft anzusehen ist, je weniger plausibel und unbegründeter sie ist, je stärker an ihr festgehalten wird, je eher sie zu Sorgen und Stressbelastung führt, und je häufiger sich die betroffene Person mit ihr beschäftigt.

In Abgrenzung zu allgemeiner Wahnsymptomatik definierten Freeman und Garety (Freeman und Garety 2000, 2014) paranoiden Wahn durch das Vorliegen mindestens eines von 2 Kriterien:

A. Die Person nimmt an, dass ihr aktuell oder in Zukunft Schaden droht, und
B. die Person nimmt an, dass der/die Verfolger:in ihr/ihm absichtlich Schaden zufügt.

Die Tatsache, dass Wahnüberzeugungen und paranoide Wahnüberzeugungen auch in der Normalbevölkerung anzutreffen sind, führt insgesamt zu der Frage, inwiefern wahnhafte Überzeugungen und Verschwörungstheorien voneinander abzugrenzen sind. Neigen ähnliche Personen in der Normalbevölkerung sowohl dazu, paranoide Wahnüberzeugungen zu entwickeln, und sind dieselben Personen auch Verschwörungstheorien eher zugeneigt?

## Zusammenhänge zwischen Verschwörungstheorien und Paranoia

Einige Untersuchungen thematisierten Zusammenhänge zwischen Paranoia und Verschwörungsglauben, beispielsweise eine Studie von Brotherton und Esser, die nachwies, dass Paranoia den Zusammenhang zwischen einer Neigung, schneller Langeweile zu empfinden und Verschwörungstheorien zu bejahen, mediierte (Brotherton und Eser 2015).

Generell bestehen aber qualitative Unterschiede zwischen Verschwörungstheorien und paranoiden Wahnüberzeugungen.

Verschwörungstheorien sind dabei:

- eher unpersönlich formuliert; die Bedrohung ist auf eine Gruppe oder die Gesamtgesellschaft ausgerichtet.
- Die Theorie und ihre Realisierung bzw. die Gefahr, die von ihr ausgeht, lässt sich durch das Sammeln von Beweisen und die Aufklärungsarbeit abwenden.
- Die verschwörungsgläubige Subkultur bietet eine große soziale Vernetzung, soziale Aufwertung und Anerkennung und nach Berichten eine ausgeprägte Toleranz gegenüber sich widersprechenden Theorien, deren Anhänger trotzdem in die Gruppen aufgenommen werden.

Paranoider Wahn zeichnet sich hingegen dadurch aus, dass:

- man selbst als Person bedroht ist.
- Menschen, die unter paranoiden Wahnüberzeugungen leiden, gelingt es in den meisten Fällen nicht, andere Menschen von ihrem paranoiden Wahn zu überzeugen (außer in den seltenen Fällen, in denen Paare oder Lebensgemeinschaften eine „folie à deux“ ausbilden, einen geteilten gemeinsamen Wahn).

Gleichzeitig sollte beachtet werden, dass Personen, die paranoiden Wahnüberzeugungen zuneigen, natürlich auch Verschwörungstheorien als realistisch ansehen können. In Onlinestudie stimmten beispielsweise Personen, die paranoide Wahnüberzeugungen aufwiesen, ebenfalls verstärkt Verschwörungstheorien zu (Bell und Raihani 2022; Greenburgh et al. 2019).

Imhoff und Lamberty fassten in ihrer Metaanalyse insgesamt 11 Studien zu Zusammenhängen zwischen Verschwörungstheorien und paranoidem Wahn zusammen und konnten einen moderaten Zusammenhang zwischen beiden Konstrukten nachweisen (*z* = 0,38) (Imhoff und Lamberty 2018). Gleichzeitig belegten sie in einer Faktorenanalyse, dass es sich bei paranoiden Wahnüberzeugungen und dem Glauben an Verschwörungstheorien um zwei distinkte Konstrukte handelt, die klar voneinander abgrenzbar sind und unterschiedliche Assoziationsmuster zu anderen psychologischen Variablen aufweisen: Paranoia stand dabei eher im Zusammenhang mit spezifischen Persönlichkeitsmerkmalen wie Introversion und Neurotizismus und einer Neigung, sich verstärkt durch andere Menschen bedroht zu fühlen. Die Neigung, Verschwörungstheorien zuzustimmen, war weniger mit Persönlichkeitseigenschaften und eher mit politischen Einstellungen wie generell geringem Vertrauen in Regierungen assoziiert.

Trotzdem bestehen selbst bei Personen, die zu sowohl paranoiden Wahnüberzeugungen als auch zum Glauben an Verschwörungstheorien neigten, Unterschiede zu Personen, die nur Verschwörungstheorien zustimmten: Personen, die sowohl zu Paranoia als auch zu Verschwörungstheorien neigten, bejahten eher Verschwörungstheorien, die mit einer persönlichen intendierten Gefahr für ihre eigene Person verbunden waren (Bell und Raihani 2022; Greenburgh et al. 2019), während Personen, die primär Verschwörungstheorien zustimmten und keine paranoide Wahnüberzeugungen berichteten, eher von einer Gefahr für die Allgemeinbevölkerung ausgingen.

## Entstehungs- und aufrechterhaltende Modelle von Paranoia und Wahn: Ist ein Transfer in den Bereich der Verschwörungstheorien sinnvoll?

In den letzten Jahrzehnten wurden in Bezug auf die Entstehung und Aufrechterhaltung von Wahnüberzeugungen bei Personen mit psychotischen Störungen sehr interessante kognitionspsychologische Modelle entwickelt: Modelle für Wahn und Halluzinationen (Bentall et al. 2014; Garety et al. 2007, 2001), Modelle für Verfolgungswahn (Freeman 2007; Freeman und Garety 2014; Freeman et al. 2002; Preti und Cella 2010) und für Negativsymptomatik (Beck et al. 2011).

**Figure 2:**
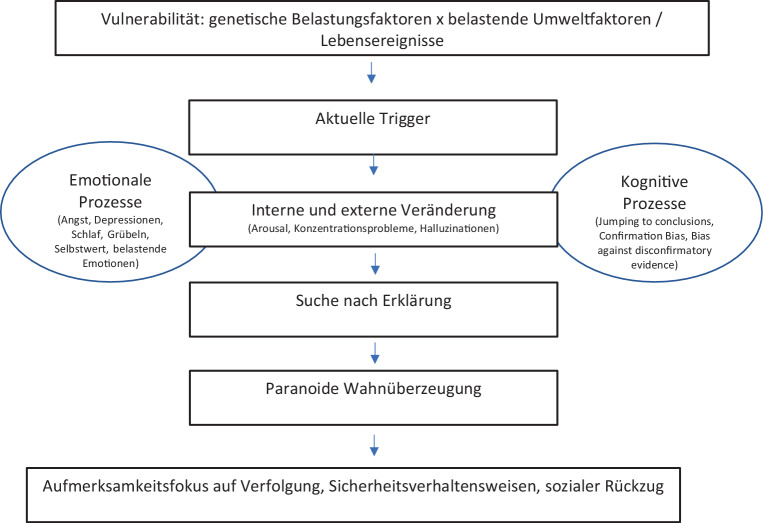

Gemeinsam ist den Modellen die Annahme, dass biologische Vulnerabilitätsfaktoren in Interaktion mit belastenden Lebensereignissen, sozialen Bedingungen und aktuellen Auslösern/Triggern zu einer erhöhten Stressbelastung führen, durch die erste Prodromalsymptome wie Wahrnehmungsveränderungen (Halluzinationen), erhöhtes Arousal oder Konzentrationsprobleme ausgelöst werden. Individuen versuchen in einem nächsten Schritt, sich die ungewöhnlichen Wahrnehmungserlebnisse zu erklären und diese zu bewerten. Dieser Bewertungsprozess wird auf der einen Seite kausal durch bestimmte kognitive Verarbeitungsstile, auf der anderen Seite durch emotionale Prozesse mediiert bzw. beeinflusst. Die Wahnüberzeugung stellt das Endresultat dieses Erklärungsversuchs da (Abb. 2).

Zu den kognitiven Verarbeitungsstilen wird z. B. die Tendenz von Personen mit psychotischen Störungen und Wahn gerechnet, wenig Informationen zu sammeln, sondern schnelle Schlussfolgerungen zu treffen, der „jumping to conclusions (JTC) bias“ (Huq et al. 1988), der in Metaanalysen bei Patient:innen mit Psychosen stärker ausgeprägt ist als bei gesunden Kontrollproband:innen (Dudley et al. 2015). Eine weitere Denkverzerrung ist der „bias against disconfirmatory evidence“ (BADE), die Tendenz, Informationen, die der eigenen Überzeugung widersprechen, nicht mehr wahrzunehmen (Moritz und Woodward 2006). Im Bereich der emotionalen Faktoren werden Angst und andere belastende Emotionen, Depressionen, negativer Selbstwert und negative Selbst- und Fremdschemata, Probleme in der Emotionsregulation sowie Grübeln und Schlafprobleme diskutiert (Fowler et al. 2012; Kesting und Lincoln 2013; Krkovic et al. 2020; Ludwig et al. 2019; Schlier et al. 2019). Zu den aufrechterhaltende Faktoren von Wahnüberzeugungen werden in den theoretischen Modell sozialer Rückzug, Sicherheitsverhaltensweisen und Bedrohungsmonitoring gerechnet.

Interessanterweise finden sich in Bezug auf die kognitiven Prozesse und emotionalen Faktoren einige Parallelen zwischen Personen, die paranoiden Wahnüberzeugungen zuneigen, und Personen, die Verschwörungstheorien anhängen. Auf diese Parallelen möchten wir im nächsten Abschnitt näher eingehen.

## Ähnliche Entstehungs- und aufrechterhaltende Bedingungen von paranoiden Wahnüberzeugungen und Verschwörungstheorien?

Trotz eindeutiger Unterschiede zwischen einer paranoiden Wahnüberzeugung im Rahmen einer behandlungsbedürftigen psychotischen Störung und dem Glauben an eine Verschwörungstheorie, der, für sich betrachtet, keine Behandlungsindikation darstellt, bestehen auch einige interessante Gemeinsamkeiten zwischen beiden Personengruppen, die möglicherweise zu der Hypothese führen könnten, dass ähnliche kausale Faktoren an der Entstehung und Aufrechterhaltung von paranoiden Wahnüberzeugungen und Verschwörungsglauben beteiligt sind.

Interessanterweise spielen sowohl bei paranoiden Wahnüberzeugungen als auch bei der Entstehung von Verschwörungsglauben in Korrelationsstudien belastende Lebensereignisse eine Rolle. Belastende Lebensereignisse wie Traumatisierung, Migration, Mobbing und Gewalterfahrungen treten gehäuft bei Personen auf, die paranoide Wahnüberzeugungen im Rahmen einer psychotischen Störung entwickeln (van Os et al. 2010). Auch bei der Entstehung von Verschwörungstheorien konnte eine Studie einen Zusammenhang zwischen belastenden Lebensereignissen, die nach Berichten der Probanden 6 Monate vor der Datenerhebung stattgefunden hatten, erhöhtem aktuell wahrgenommenen Stress und einer Zustimmung zu Verschwörungstheorien nachweisen (Swami et al. 2016b). Weitere Zusammenhänge betreffen eher gesellschaftliche oder politische Ereignisse, die als ursächlich für Verschwörungstheorien diskutiert werden und häufig viele Menschen betreffen, wie beispielsweise soziale Ungleichheit, gesellschaftliche Krisen, undurchsichtiges Verhalten von Autoritäten, Polarisation und Misinformation (Douglas et al. 2019; Uscinski et al. 2016; van Prooijen und Douglas 2018). Ähnlichkeiten bestehen auch in einem geringen Vertrauen in andere Menschen, das im Zusammenhang zur Zustimmung zu Verschwörungstheorien steht (Abalakina-Paap et al. 1999; Klein et al. 2015), aber auch mit der Zustimmung zu paranoiden Wahnüberzeugungen (Fenigstein und Vanable 1992) assoziiert ist.

Es finden sich ebenfalls externale Kontrollüberzeugungen und ein externalisierender personalisierender Kausalattributionsstil: bei Personen, die Verschwörungstheorien zustimmen (Douglas et al. 2016; Suthaharan et al. 2021; Van Prooijen und Acker 2015; van Prooijen und Douglas 2018), und auch bei Personen mit psychotischen Störungen und paranoiden Wahnüberzeugungen (Lincoln et al. 2010; Mehl et al. 2015).

Die Entwicklung einer Verschwörungstheorie oder einer paranoiden Wahnüberzeugung basiert ebenfalls häufig auf der Wahrnehmung und Gewichtung von verschiedenen sich teilweise widersprechenden Informationen aus der Umgebung und auf entsprechenden Schlussfolgerungen. Interessanterweise machten hier Personen, die Verschwörungstheorien bejahten, in einer experimentellen Beurteilungsaufgabe etwas häufiger Fehler als Kontrollprobanden (Brotherton und French 2014). Auch Patient:innen mit paranoiden Wahnüberzeugungen wiesen in einer Metaanalyse über 55 Studien in einer Entscheidungsaufgabe (Beads task: Huq et al. 1988) eine Neigung auf, im Vergleich zu gesunden Kontrollproband:innen voreiligere Schlussfolgerungen zu treffen und dabei weniger Informationen zu sammeln (Dudley et al. 2015). In einer Online-Studie wiesen wir ebenfalls nach, dass Personen, die in einer ähnlichen Entscheidungsaufgabe (Fische-Aufgabe: basierend auf geangelten Fischen in bestimmten Farben werden Proband:innen gebeten, zu entscheiden, in welchem der zwei Fischteiche die Fische gefangen wurden) voreilig und zu früh entschieden, eher Verschwörungstheorien zustimmten als Personen, die mehr Informationen einholten und länger abwarteten, bevor sie eine Entscheidung trafen; wir konnten ebenfalls einen mittleren Effekt des Gruppenunterschieds nachweisen (*d* = 0,53: Pytlik et al. 2020).

## Fazit

Auch wenn zwischen Personen, die Verschwörungstheorien, und Personen, die paranoiden Wahnüberzeugungen (beispielsweise im Rahmen einer psychotischen Störung) zustimmen, einige Parallelen bestehen, so unterscheiden sich beide Gruppen natürlich in sehr vielen anderen psychologischen Faktoren. Dabei ist der wichtigste Faktor, dass Personen mit paranoiden Wahnüberzeugungen meistens (nicht immer) unter einer behandlungsbedürftigen psychischen Störung leiden, während eine solche bei Personen, die an Verschwörungstheorien glauben, meist nicht vorliegt.

Nichtsdestotrotz bestehen interessante Parallelen zwischen beiden Gruppen. Diese beziehen sich auf Zusammenhänge zwischen belastenden Lebensereignissen bzw. Umweltfaktoren, die eine Rolle in der Entstehung beider Faktoren spielen könnten. Beide Personengruppen berichten ebenfalls von einem geringeren Vertrauen in andere Menschen, und beide Gruppen nehmen an, dass Lebensereignisse weniger ihrer eigenen Kontrolle, sondern eher der Kontrolle äußerer Faktoren oder anderer Personen unterliegen (externale Kontrollüberzeugungen, external personalisierender Kausalattributionsstil). Beide Personengruppen weisen im Vergleich zu gesunden Kontrollproband:innen eine Neigung auf, Schlussfolgerungen schneller zu treffen und dabei weniger Informationen zu sammeln, bevor sie zu einer Entscheidung gelangen. Es ist an dieser Stelle zu beachten, dass viele dieser Befunde auf nicht präregistrierten Studien basieren, es sich also auch um Zufallsbefunde handeln könnte. Nichtsdestotrotz sind mögliche Parallelen zwischen beiden Gruppen sehr spannend.

Sollten sich die Hinweise auf ähnliche kognitionspsychologische Mechanismen bei Personen mit Verschwörungstheorien und paranoiden Wahnüberzeugungen verdichten, ist zunächst die Frage zu stellen, ob eine solche Ähnlichkeit tatsächlich auf ähnliche kausale Prozesse hinweist.

Für die Annahme einer Kausalität zwischen einem Faktor und einer Wirkvariable sollten nach Schwartz und Susser (2006) 3 Bedingungen erfüllt sein: 1) eine *Assoziation* zwischen einem möglichen kausalen Faktor und der Wirkvariable, 2) *Zeitreihenfolge*: der potenzielle kausale Faktor sollte zeitlich der Wirkvariable vorangehen, und 3) *Richtung*: eine Veränderung in dem möglichen kausalen Faktor sollte im Zusammenhang mit der Wirkvariablen stehen. Im Bereich der kognitionspsychologischen Modelle in Bezug auf die Entstehung und Aufrechterhaltung von Wahn ist die Annahme von Kausalität nicht immer zweifelsfrei nachgewiesen (für eine Zusammenfassung siehe: Mehl et al. 2018).

Bezüglich eines möglichen Transfers der Modelle in den Bereich der Entstehung und Aufrechterhaltung von Verschwörungstheorien sind die vorliegenden Befunde noch sehr lückenhaft, und in den meisten Fällen ist lediglich die erste Bedingung (Assoziation) hinreichend erfüllt. Auch werden in den kognitionspsychologischen Modellen der Entstehung und Aufrechterhaltung von Wahnüberzeugungen Umweltbedingungen und politische Bedingungen stark unterschätzt, die Eingang in Modelle finden sollten, die die Entstehung und Aufrechterhaltung von Verschwörungstheorien zu erklären versuchen.

Somit besteht ein erster wichtiger Schritt darin, mögliche längsschnittliche und kausale Assoziationen zwischen den kausalen Faktoren und paranoiden Wahnüberzeugungen und Verschwörungstheorien weiter zu prüfen (basierend auf präregistrierten Studien). In einem ersten Schritt sollten zunächst weitere Ähnlichkeiten auf längsschnittlicher und experimentalpsychologischer Ebene im Bereich der paranoiden Wahnüberzeugungen und im Bereich der Verschwörungstheorien nachgewiesen werden. Aus einem solchen Nachweis ergeben sich weitere interessante Fragestellungen, beispielsweise bezüglich relevanter Unterschiede, die die Entwicklung einer Verschwörungstheorie oder einer paranoiden Wahnüberzeugungen begünstigen könnten.

Aufgrund der Dringlichkeit des gesellschaftlichen Problems der Verschwörungstheorien wäre es gleichzeitig ratsam, empirisch zu prüfen, ob Interventionsmethoden, die bei Patient:innen mit paranoiden Wahnüberzeugungen wirksam sind, möglicherweise auch für Personen hilfreich sein könnten, die Verschwörungstheorien zuneigen und motiviert sind, ihre Neigung zu verändern. Die Interventionen könnten beispielsweise den Betroffenen in Beratungsgesprächen als Präventionsmaßnahme angeboten werden. Dabei ist zu natürlich beachten, dass Personen mit einer Neigung zu Verschwörungsglauben meist nicht psychisch erkrankt sind. Somit ist ein niedrigschwelliger Zugang zu Beratungsangeboten sehr wichtig. Gleichzeitig finden viele Interventionen der Kognitiven Verhaltenstherapie auch bei der Prävention von psychischen Krisen und Erkrankungen Anwendung.

Als mögliche Interventionsmethoden wären Methoden der Kognitiven Verhaltenstherapie bei Psychosen und Wahn zu nennen, die in aktuellen Behandlungsleitlinien (DGPPN 2019; Lincoln et al. 2019) als wirksam in Bezug auf die Reduktion von Symptomen eingestuft wurden. Beispielsweise könnten Personen, die zu Verschwörungsglauben neigen, trainieren, ihre automatischen Gedanken und Bewertungen von Situationen zu überprüfen und dabei weniger logische Fehler zu machen (weitere Interventionen sind bei Mehl und Lincoln 2014 beschrieben). Ein weiteres niedrigschwelliges Programm ist das Metakognitive Training (MKT; Moritz und Woodward 2007). Im Rahmen des MKT werden Patient:innen über ungünstige Folgen ihrer Tendenz, voreilige Schlussfolgerungen zu treffen, informiert. Sie trainieren anschließend gezielt, vorsichtigere Schlussfolgerungen zu treffen und gezielter Informationen zu sammeln, bevor sie über einen Sachverhalt entscheiden. Das Training war in Metaanalysen im randomisierten kontrollierten Design ebenfalls erfolgreich in der Reduktion von Wahn und der Reduktion der Tendenz, voreilige Schlussfolgerungen zu treffen (Philipp et al. 2019). Insgesamt bestehen in der Untersuchung von Ähnlichkeiten und Unterschieden von Verschwörungstheorien und paranoiden Wahnüberzeugungen in subklinischen und klinischen Gruppen interessante Möglichkeiten, ein besseres Verständnis beider Konstrukte zu entwickeln und Interventionsmöglichkeiten in diesem Bereich weiterzuentwickeln.
